# Technical trick: buttress plate fixation of comminuted capitellum fractures with combined suture repair of the lateral ulnar collateral ligament

**DOI:** 10.1016/j.xrrt.2023.06.004

**Published:** 2023-07-28

**Authors:** Urvi J. Patel, Akhil Dondapati, Thomas Carroll, Sandeep Soin

**Affiliations:** University of Rochester Department of Orthopaedic Surgery, University of Rochester Medical Center, Rochester, NY, USA

**Keywords:** Capitellum fracture, Lateral ulnar collateral ligament repair, Buttress plating, Lateral column repair, Elbow, Distal humerus

Fractures of the capitellum represent 1% of all elbow fractures and 6% of all distal humerus fractures.^1^^,^^7^ Injuries typically occur with low-energy falls onto an outstretched hand, direct axial compression of the distal humerus with the elbow in a hyperextended or semiflexed position, or with posterolateral elbow dislocations. These capitellum fractures have an associated radial head and/or lateral ulnar collateral ligament (LUCL) injury 60% of the time and are commonly associated with elbow fracture-dislocations.^2^^,^^9^^,^^12^^,^^13^

No gold standard currently exists for fixation of capitellum fractures. Previously described methods include metallic screws, bioabsorbable screws, Kirschner wires, fragment excision, and fibrin glue.^3^^,^^15^^,^^16^ Headless compression screws have increasingly become the most commonly used fixation technique. Anterior buttress plating has recently been described as a biomechanically superior fixation technique with significantly higher load-to-failure rates.^11^ LUCL injuries are most commonly repaired using standard bone tunnels or suture anchors; however, in the setting of concomitant capitellum fractures, this can be challenging due to fragmentation of the central point of the lateral elbow, the usual anchor location.^4^, ^5^, ^6^^,^^8^

An alternative fixation method, described in this paper, combines lateral buttress plating with suture fixation of the LUCL to the buttress plate construct as an anchor point. Bone tunnels and suture anchors rely on adequate bone stock for fixation, which is frequently absent in the setting of comminuted capitellum fractures with ligamentous injuries. The fixation method described here allows for adequate stabilization of the fracture, as well as the LUCL, in the absence of a viable bone bed for anchor placement.

This study seeks to describe a novel surgical technique combining lateral column buttress plating of comminuted capitellum fractures with suture fixation of the LUCL. A detailed description of this technical trick can provide surgeons with an alternative fixation method for capitellum fractures with LUCL injuries that may demonstrate improved clinical outcomes.

## Technique

**Figure 1:**
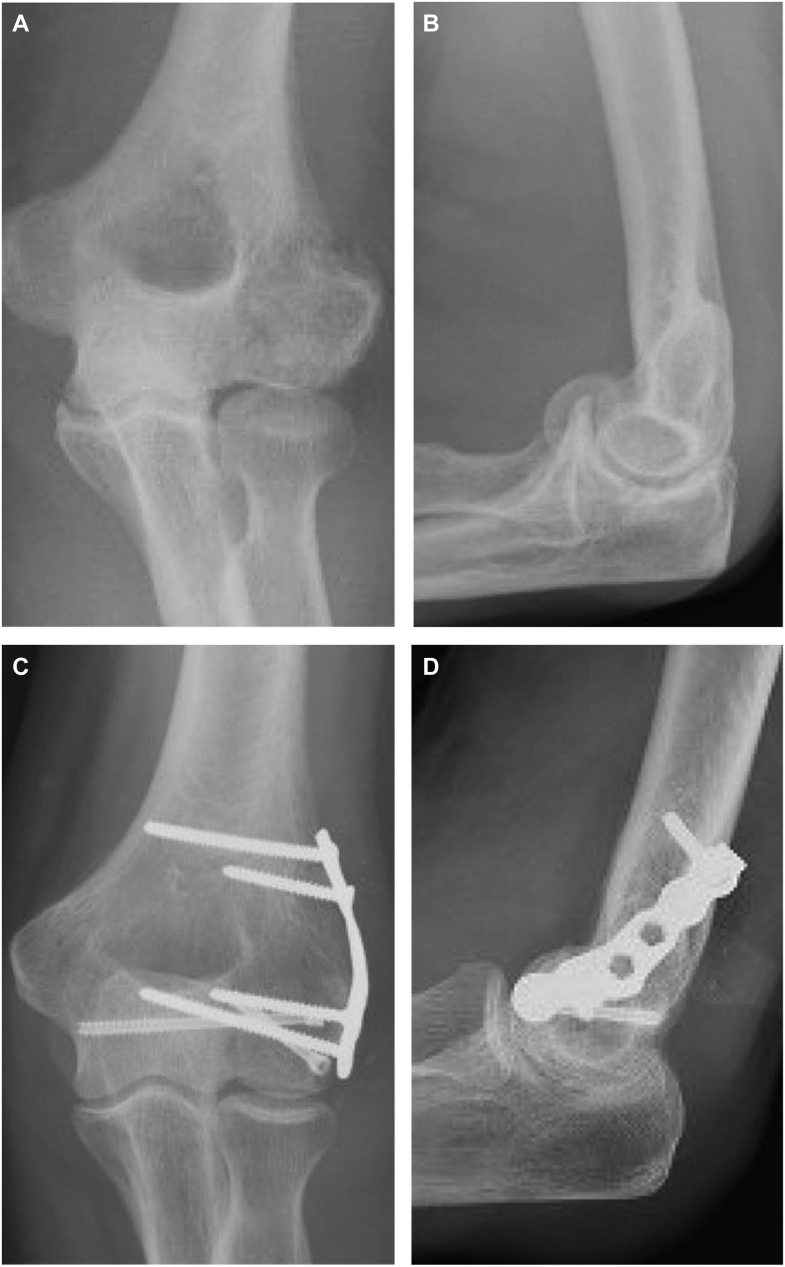
Radiographic images of a comminuted capitellum fracture (**A** and **B**) treated with headless compression screws and buttress plating (**C** and **D**).

In the setting of complex fracture-dislocations of the elbow with comminuted capitellum fractures [Fig. 1, *A* and *B*], x-ray imaging and computed tomography scans can be useful in assessing intra-articular fragments, concentric reduction of the radiohumeral and ulnohumeral joints, and fracture extension into the trochlea. These findings guide surgical planning and operative fixation.

Our preference is for fixation in the supine position with use of an arm board attachment to the operative table. Although complete skeletal paralysis is not always indicated, it may be helpful for difficult reductions and to assist with achieving length and allowing for manipulation at the fracture site. Transfer the patient to the edge of the bed of the operative extremity to guarantee ample shoulder mobility, allowing manipulation of the arm as needed. The patient’s operative shoulder should lay flush with the arm table at the arm board-operative table interface. Ensure that the shoulder is not hyper abducted or hyper adducted as this may increase the risk of a postoperative brachial plexopathy. A nonsterile tourniquet is placed proximally on the arm to assist with hemostasis.

We prefer an extensor digitorum communis (EDC) split approach to the elbow. This approach allows for improved access to the anterior aspect of the capitellum while reducing the risk of iatrogenic injury to the LUCL complex if uninjured. The lateral epicondyle and radial head are palpated similar to the Kocher or Kaplan approaches, and a 7-cm oblique lateral incision is marked overlying the proximal edge of the lateral epicondyle, extending distally over the central aspect of the radial head towards the posterior ulnar border. Pronation of the forearm is utilized to protect the posterior interosseous nerve. An incision is made to the level of the fascia, and the EDC tendon is then identified anteriorly. Identify the natural split of the EDC muscle proximally and follow dissection distally in line with this plane. Take care to identify the LUCL and protect it during the entirety of the case. The joint capsule and annular ligament should now be revealed. The capsulotomy is made collinear with the EDC split just anterior to the equator of the capitellum. Extension of the approach can be made distally by incising the annular ligament if needed and proximally by releasing the extensor origin from the lateral epicondyle.

**Figure 2:**
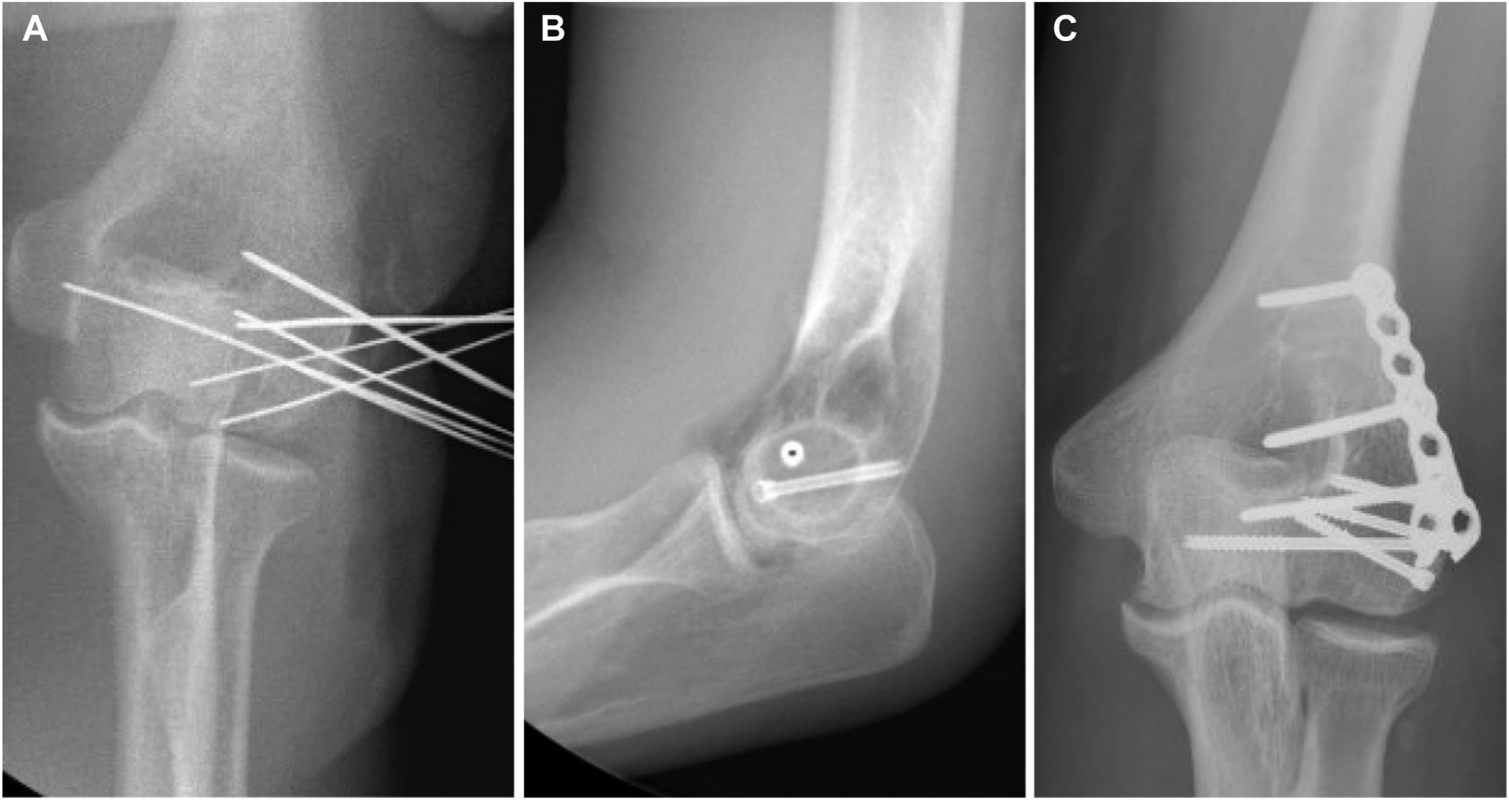
Intraoperative images of capitellum reduction with use of Kirschner wires (**A**) and placement of headless compression screws from lateral to medial and anterior to posterior (**B**), followed by the addition of a mini buttress plate over the headless compression screws (**C**).

**Figure 3:**
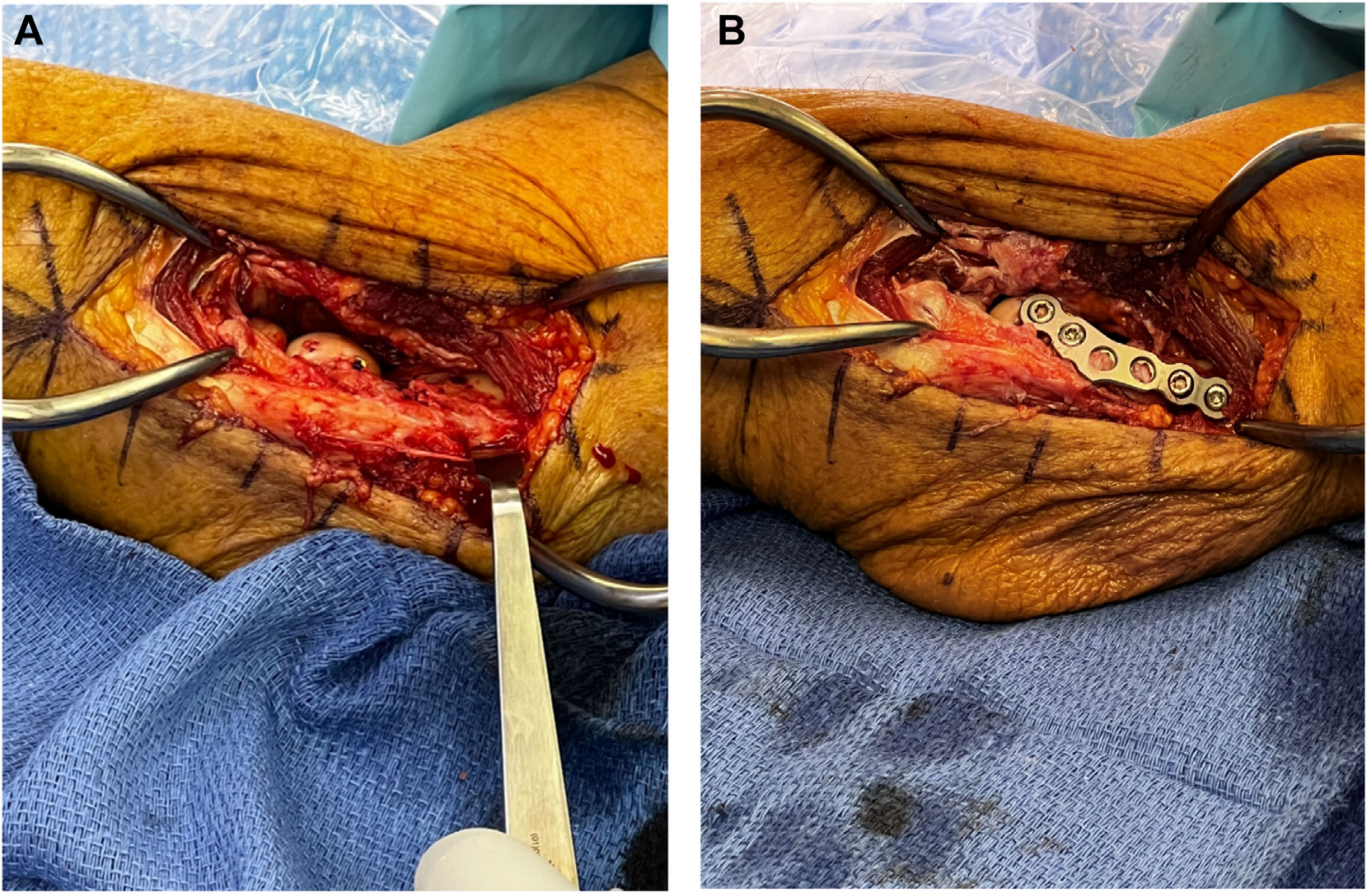
Intraoperative images of headless compression screw fixation (**A**) followed by application of mini fragment buttress plate to the lateral column (**B**).

Once the fracture fragment is identified, gently retract it anteriorly to allow for irrigation, débridement, and hematoma evacuation. Manipulate the fragment to its anatomical position with extension of the elbow to assist with reduction. Use small-diameter Kirschner wires to hold the fragments reduced to the trochlea and the lateral column [Fig. 2, *A*]. Following this, use 1.1 mm Kirschner wires, which correlate with our preferred 3.0 cannulated headless compression screws, to hold the fragment reduced to the distal end of the humerus. Our preference is for the placement of 2x 1.1-mm Kirschner wires, one from lateral to medial to allow for compression of the articular fragments and one from anterior to posterior to compress the capitellum to the shaft of the humerus [Fig. 2, *B*]. Additional 1.1-mm Kirschner wires may need to be placed if there remain any additional fragments that need fixation. Ensure appropriate reduction under fluoroscopic guidance. The Kirschner wires are over drilled through the near cortex, and the appropriately sized 3.0 mm headless compression screws are placed until flush below the articular cartilage [Fig. 3, *A*].

As previously mentioned, the two screws serve to resist fracture fragment rotation and shearing forces. However, in cases with increased comminution and poor bone stock, additional points of fixation are warranted to achieve stable osteosynthesis. Our preference is for the placement of a lateral buttress plate using a 2.7-mm mini fragment system [Figs. 2, *C* and 3, *B*]. The appropriately sized locking plate is selected and contoured to the distal lateral portion of the humerus to traverse the fragment. We recommend the use of any surgeon-preferred mini-fragment plating system. Variable-angle locking technology can be advantageous. These plates can be trimmed and contoured to appropriately fit the lateral column in buttress fashion. Our preference is for a T-shaped or L-shaped trim to the plate. Place the contoured plate along the lateral aspect of the distal humerus and confirm appropriate positioning under fluoroscopic guidance. The plate is typically compressed to the bone above the level of the comminution to compress and capture the fragmented lateral column. In the setting of lateral epicondyle avulsion, the plate can serve to capture and secure the origin of the LUCL. Locking screws can often be placed in the most distal aspect of the plate, neutralizing forces on the articular repair. Ensure appropriate placement of the implants as well as the anatomic position of the fragment under fluoroscopic guidance [Fig. 1, *C* and *D*].

At this point, it is important to ensure stability of the elbow joint with varus and valgus stress. With avulsion of the LUCL, there will be instability with varus stress that must be appropriately addressed. With a comminuted lateral column, bone tunnels and anchor placement become difficult due to failure of purchase in poor bone stock. Our preference is to repair or reinforce the LUCL to the plate using #2 suture strong, braided nonabsorbable suture in a running locking fashion. Pass the suture multiple times through the plate along the lateral column. Following this, incorporate the LUCL and lateral ligament complex into the suture in a running whip stitch manner along the length of the ligament. The isometric point is estimated based on plate placement and with intraoperative examination of the elbow in flexion, extension, pronation, and supination. The authors prefer LUCL fixation to be 2 mm more anterior and 2 mm more proximal than the classically described anatomic origin at the center of the capitellum. This allows us to slightly overtension the complex. By moving the anchor point off of the anatomic isometric point, the native fibers are able to abut the anatomic position or be slightly overtensioned. We recognize that this may result in a small loss of elbow extension; however, there is the added benefit of increased stability. With the use of the mini fragment plate as an adjunct for LUCL repair, the plate is contoured and placed in a position that allows for an anchor point that is positioned slightly more anterior and more superior. With the vast variety of mini-fragment plates on the market, the subtle differences in plate hole placement can act as different anchor points, which allow for adjustment of suture passage and ultimate anchorage. The suture ends are then tied, and stability is evaluated through range of motion (ROM) and a stress examination.

After confirmation of appropriate reduction and implant placement in multiple projections, the incision is irrigated and closed in layers. We prefer limited immobilization in bulky cotton dressing. Patients remain immobilized in the bulky dressing for approximately one week, then are transitioned to ROM to tolerance. Patients remain non-weight bearing for a total of 6 to 8 weeks, with progressive resistance and weight bearing thereafter.

## Case series

After the institutional review board approval, patients with comminuted capitellum fractures treated with buttress plating by a single surgeon were retrospectively reviewed. Patients over the age of 18 who sustained the aforementioned injury in isolation were included in the study. Patients under the age of 18 or polytrauma patients were excluded from the study. Patient demographics, weight-bearing status, and radiographic and clinical outcomes were collected.

All patients sustained displaced comminuted capitellum fractures. Four of the 6 patients also underwent repair of the LUCL. Fractures were further classified by the Bryan and Morrey classification system. Final follow-up radiographs were evaluated for union.

Six patients, each with a comminuted capitellum fracture and four with associated LUCL injury or avulsion fractures, were identified [Table I]. All cases were managed by a single fellowship-trained, board-certified orthopedic trauma surgeon. The average patient age was 51.5 years. Five patients were female (83%), and 1 patient was male (17%). All six patients had computed tomography scans performed prior to surgical intervention. All six patients had a mechanism of injury consistent with a fall onto an outstretched arm. Two patients had Bryan and Morrey -classified fracture type III (33%), 1 patient had type IV (17%), and three patients had type I (50%). Two patients sustained a fracture-dislocation during the initial injury. We had an average postoperative follow-up of approximately 6 months. Of the 6 patients who underwent our preferred surgical technique, none required revision surgery. Two patients had minor radiographic evidence of heterotopic ossification along the LUCL, however this was largely asymptomatic. All six patients were kept partially immobilized for 1 week, followed by removal and ROM exercises as tolerated. All six remained nonweight bearing for approximately 8 weeks.

**Table I:** Patient demographics, outcomes, and complications.

Age	Gender	BMI	MOI	Fracture-dislocation?	Preoperative nerve sxs?	Bryan and Morrey classification	Dubberley classification	LUCL repair?	WB status	Follow-up (months)	Elbow ROM at final follow-up	Complications
57	F	31	FOOSH	Yes	Yes, resolved w/ Reduction	Type III	Type Ib	Yes	NWB x 8 wks	14	30–140, full pronation/ supination	None
69	F	30	FOOSH	No	None	Type IV	Type IIa	Yes	NWB x 8 wks	6	15-140, Full pronation/ supination	HO along LUCL
32	M	35	FOOSH	No	None	Type I	Type Ia	Yes	NWB x8 wks	2	30-140, Full pronation/ supination	None
63	F	30	FOOSH	Yes	Baseline anterior interosseous nerve deficits	Type III	Type Ib	Yes	NWB x8 wks	11	40–100, full pronation, limited supination	HO along the LUCL, ulnar nerve neuropathy 5 mon after sx
59	F	18	FOOSH	No	None	Type I	Type Ia	No	NWB x8 wk	4	10–130, full pronation/ supination	None
29	F	31	FOOSH	No	None	Type I	Type Ia	No	NWB x6 wks	1	10–125, full pronation/ supination	None

*NWB*, non-weight bearing; *WB*, weight bearing; *HO*, heterotopic ossification; *LUCL*, lateral ulnar collateral ligament; *ROM*, range of motion; *MOI*, mechanism of injury; *FOOSH*, fall on an outstretched hand; *BMI*, body mass index.

None of the patients had to return to the operating room for revision procedures. There were no cases of postoperative instability. There were no cases of deep or superficial infection. With the exception of one patient who underwent complex revision surgery, the average elbow ROM with extension and flexion at final follow-up was 19–135 degrees. Six patients had full pronation, and 5 patients had full supination. One patient with full pronation and full supination had an initial injury consistent with a fracture-dislocation of her elbow and the need for medial ulnar collateral ligament repair intraoperatively.

One patient received our preferred fixation as a salvage procedure after a failed open reduction and internal fixation. This patient had baseline anterior interosseous nerve deficits and a complication of ulnar nerve neuropathy 5 months after surgery. While she underwent anterior interosseous nerve and ulnar nerve decompression surgery, her elbow remained stable and symptom-free. She was able to progress with physical therapy and denied any instability in the elbow. Her elbow ROM was 40–100 degrees with full pronation and 70 degrees supination at the most recent follow-up. This patient presented as an outlier as she was a revision surgery.

## Discussion

Our series shows encouraging results with open reduction and internal fixation with buttress plating and concomitant repair of the LUCL in patients with comminuted capitellum fractures. All of our patients sustained their injury after low-energy falls onto an outstretched arm. There were no cases of revision surgery, and all patients with follow-up returned to activity as tolerated.

The primary advantage of our technique is that it allows for added stability to the overall construct through the use of a buttress plate. Additionally, we provide an alternative to LUCL repair without the need for suture anchor fixation, which becomes technically challenging in cases of comminuted capitellum fractures with poor lateral column bone stock. While prior biomechanical studies have described the advantages of anterior buttress plating in addition to headless compression screws,^11^ none to our knowledge have described lateral column buttress plating or an alternative technique to LUCL avulsion repair in cases with poor lateral column bone stock.

Nolte et al recently described an anterior buttress technique in addition to headless compression screws for fixation of capitellum fractures.^11^ Their biomechanical study showed significantly higher load-to-failure rates for 2-mm fragment displacement in cadaveric specimens with headless compression screws with an added anterior buttress plate compared to headless compression screws alone. However, all cadaveric specimens were Dubberley type IA shear fractures of the capitellum, which limits the generalizability of their outcomes.

Bryan and Morrey type III fractures are more complex with comminution of the lateral column, making it difficult to obtain adequate reduction and fixation of the fracture. While type I, II, and IV fractures have an intact posterolateral column, osteoporotic and traumatized bone may make adequate fixation technically challenging. Mighell et al describe the addition of a posterolateral small fragment buttress plate in cases where a stable bony bed for reattachment of the articular fragments is not present.^10^ This, however, is a larger implant that would require extension of the lateral incision and soft tissue dissection posteriorly for plate placement. Additionally, their method does not address avulsion fractures from LUCL origin in these cases of comminuted capitellum fractures.

The lateral collateral ligament complex serves as a primary stabilizer of the elbow joint, particularly in resistance to posterolateral rotational forces and varus instability. Injury to the lateral soft tissue complex after elbow trauma is common, and restoration of the lateral ligamentous complex is critical for functional outcomes. While no gold standard exists, the most common approach to LUCL repair is with the use of suture anchors or bone tunnels. However, in cases of comminuted capitellum fractures, direct repair of the ligament is not feasible given the poor lateral column bone stock, and alternative methods must be sought out to address elbow stability.

Oftentimes, alternatives considered include ligament reconstruction with allograft or autograft or the placement of an external fixator. However, these surgical alternatives are associated with complications such as elbow stiffness, donor site pain, and infection.^14^ Our technical trick with the repair of the LUCL to the buttress plate in a running-locking fashion shows promise for favorable functional outcomes. The buttress plate serves as an anchor point in the setting of poor lateral column bone stock for the LUCL. In cases where LUCL-origin avulsion fractures go unrecognized and unrepaired, elbow instability remains, leading to poor postoperative functional outcomes.

**Figure 4:**
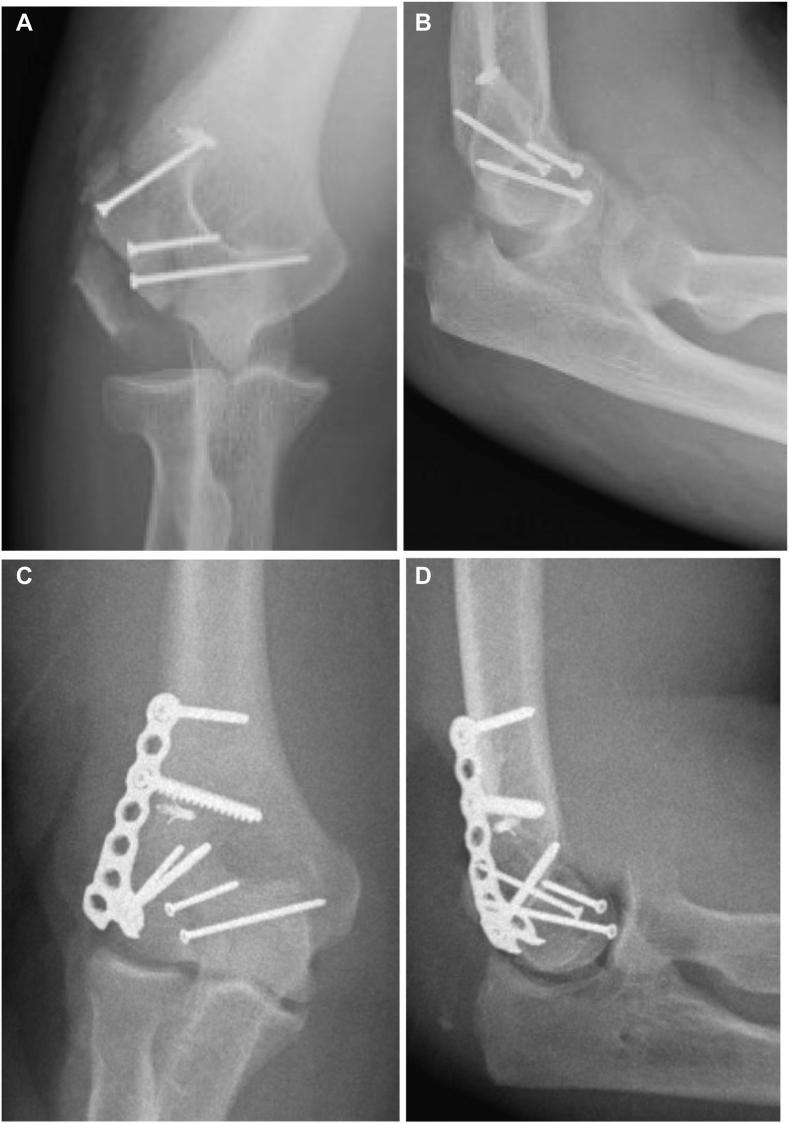
Example of a patient with failure of the LUCL origin avulsion suture anchor repair (**A** and **B**) and subsequent salvage procedure for the unstable elbow and failed fixation construct with the addition of a lateral mini fragment buttress plate and repair of the LUCL using the buttress plate as an anchor point (**C** and **D**). *LUCL*, lateral ulnar collateral ligament.

In our case series, the 63-year-old female patient is a prime example of the importance of LUCL repair. This patient initially underwent open reduction and internal fixation for a Bryan and Morrey type III capitellum fracture with headless compression screws and repair of the LUCL with suture anchor fixation. Two-week follow-up films showed catastrophic failure of the construct, with postoperative elbow instability and subsequent elbow dislocation. The patient returned to the operating room for revision fixation of the fracture with the addition of a lateral buttress plate and repair of the prior failed LUCL avulsion repair using the buttress plate as an anchor point. This salvage procedure proved to be a stable construct [Fig. 4].

There are a few limitations to our study. This is a retrospective chart analysis, which can introduce selection bias. Additionally, we have limited follow-up for a few of our patients, which limits the interpretation of our findings. Patients who failed to present for their postoperative clinical appointments may have unknown complications that are unable to be ruled out due to the limited time frame for follow-up.

## Conclusions

We provide an alternative for fixation of comminuted capitellum fractures with an associated LUCL avulsion fracture in the setting of poor lateral column bone stock without the associated complications often seen with prior described methods. Our findings demonstrate that in patients with comminuted capitellum fractures with an associated LUCL avulsion, repair to the buttress plate as an anchor point restores varus stability to the elbow. Ultimately, our mini-fragment lateral column buttress plating and LUCL repair technique is an effective method for stable fracture fixation of comminuted capitellum fractures and restoration of ligament integrity.

## Disclaimers:

Funding: No funding was disclosed by the authors.

Conflicts of interest: Sandeep Soin is a paid consultant for Skeletal Dynamics. All the other authors, their immediate families, and any research foundations with which they are affiliated have not received any financial payments or other benefits from any commercial entity related to the subject of this article.
